# The Voluntary Hospitals and Insured Persons

**Published:** 1912-11-09

**Authors:** 


### The Hospital

The Workers' Newspaper of Administrative Medicine and Institutional Life, Administration, National Insurance and Health.

No. 1375, Vol. LIII. SATURDAY, NOVEMBER 9, 1912. PRICE ONE PENNY.

### THE VOLUNTARY HOSPITALS AND INSURED PERSONS.

It is impossible for the managers of the voluntary hospitals to delay longer a definite decision as to the course they will pursue in regard to the admission or non-admission of insured persons to free in- and out-patient treatment at their institutions. After January 15, 1913, it is possible that in many voluntary hospitals quite half the patients who apply for admission may be insured persons. At one great hospital, which has been taking a census and keeping an accurate record of the in-patients admitted to treatment since the National Insurance Act came into force, it has been ascertained that upwards of 45 per cent. of the in-patients are insured persons, and that in the maternity wards 70 per cent. of the patients are insured. We are fully aware that the first impression these figures may convey to an ordinary member of a hospital committee is that the wisest course will be to accept the position and continue to admit all applicants for free medical relief on the old basis, without inquiring as to whether they are insured persons or not. This seems to be the view of a member of the committee of the Warneford Hospital, Leamington, who is also vice-chairman of a local Insurance Committee, whose letter appears in another column.

It should be unnecessary to point out, though apparently it is not, that the fact is every voluntary hospital which adopts the policy of *laissez-faire* in regard to insurance patients will in all probability soon cease to have an income from voluntary sources sufficient to carry on its work. Another point which must be remembered is that the medical profession have given notice to the hospitals that the members of neither the honorary medical staff nor the paid staff can, in existing circumstances, treat as free patients any insured person at a voluntary hospital, except in case of emergency. Of course the Chancellor of the Exchequer may come to terms with the members of the medical profession before January 15 next, but if he does, unless those terms cover payments for attending patients in a voluntary hospital, the position *qua* insured persons seeking free admission to voluntary hospitals must, in regard to medical treatment, remain unchanged so far as the practitioners are concerned.

Let us, however, set aside for the moment all question of the medical treatment of insured persons seeking free relief at the voluntary hospitals. What, in fact, is the position of the managers of a voluntary hospital *vis-à-vis* with an insured person? Certainly such a person cannot in any circumstances be held to be a fit object for free relief at a voluntary hospital. In Germany the artisan class, who make similar payments under the National Insurance Acts there, have rejected free hospital treatment on the ground of its being charity, and so a degradation for them to accept. Are the working classes of the United Kingdom to show a less independent spirit, and after making their payments for adequate medical benefits under the Insurance Act, will they with whispered humility suffer the degradation refused by their fellows in Germany? Will they, again, seek free relief as charity at the voluntary hospitals throughout the United Kingdom? We do not believe they will for one moment consent to follow any such courses. If they were to follow them it is perfectly clear that the hospital managers could not consent to admit these insured workers to free hospital treatment. We say could not because, in the first place, if they did they would commit a breach of their constitution and depart from the objects for which their institutions were originally founded. Such a course would lay them open to an injunction by any intelligent governor or subscriber possessed of the public spirit exhibited by Mr. Thomas Gibson Bowles in regard to the illegal collection of the income tax.

Nor is this all, for it must be perfectly clear to every man of business who thinks the matter out clearly, that, however philanthropic a man or woman may be, being as they are mainly employers of insured persons, they must feel that as the law compels them to pay towards the cost of adequate medical treatment for those they employ, so it is essentially their duty to insist that the State shall provide the adequate medical treatment for which they and their workers have paid under the National Insurance Act. If we are right in this view, as we have good reason to believe we are, it must be plain that, should the hospital managers favour the adoption of a policy of *laissez-faire*, they will never be able to carry it out for want of the necessary funds.

Speaking roughly, the amounts contributed by employers and insured persons in Germany and in England are approximately the same. In Germany insured persons are admitted to the German hospitals on payment of three marks per diem more or less, which is paid to such hospital by the Kasse or approved society to which they belong. In England no provision has been made for hospital benefit to insured persons, and the effect of the passing of Mr. Lloyd George's National Insurance Act, as it stands to-day, is that after January 15 next thousands of members of the working classes may be unable to obtain the hospital treatment with which they have heretofore been provided by the voluntary hospitals. It is not the fault of the working classes, being insured persons, nor of the hospital managers, nor of anybody but the present Government, and especially of Mr. Lloyd George, the Chancellor of the Exchequer, that this appalling situation has been created.

The reason of this neglect to foresee and provide against a catastrophe of this kind is that sufficient allowance was not made for the fact that adequate medical treatment cannot possibly be obtained at the present time without calling in the aid on a business basis of the voluntary hospitals—a result of the hurry with which this ill-considered legislation was forced on the country. This undue haste has rendered it impossible to make the scheme workable, just and effective, before embodying it in an Act of Parliament. The plan of finance fails from the circumstance that the money which might otherwise have been available for hospital benefit has been and will be kept back for many years to come. First of all, insured persons have been deprived of all benefits for the first six months, from the date when they first began to make contributions to the Treasury. Secondly, all who join at age 16 for some twenty years to come will be deprived of a share in a sum which in the whole amounts to £60,000,000. This sum represents the State's subsidy under our National Insurance Act which is to be applied towards liquidating the estimated deficiency of over £60,000,000, arising under Mr. Lloyd George's plan of admitting persons to the benefit of national insurance at ages over sixteen at the rate of contribution applicable to persons of that age.

All interested in national insurance and all voluntary hospital managers have, therefore, to be up and doing. We have suggested the immediate summoning of representatives of all the voluntary hospitals to a great meeting in London that some definite course of action in regard to the present crisis may be agreed to, and pursued with energy, until the Government has provided a way out. If hospital managers determine, as they must determine, to perpetuate the voluntary hospital system in these islands, the course of action to adopt is plain and simple. Arrangements must be come to for the immediate amending of the National Insurance Act upon a plan which will guarantee to the working classes the hospital benefits which the German working classes receive already, upon a basis of payment which will include a sum for the provision of medical attendance and hospital care.

The way out, in our judgment, is for the voluntary hospitals to lay down a rule and to communicate it to the Government, that they are prepared to meet the difficulty by agreeing to take insured persons in the State wards for a *pro-rata* payment which will defray the whole cost entailed upon the hospitals by the admission and treatment of each insured person plus 10 per cent. for the remuneration of the medical staffs. They ought, further, to point out to the Goverment that it will be necessary to provide a great addition to the existing number of available beds in hospitals, and that the right and most economical way of making such provision will be to empower the Local Government Board to make loans to hospital authorities who are prepared to extend the area of their work by erecting new pavilions upon sites already existing or to be acquired. The basis of such loans should be, interest at 3 per cent. per annum, plus an additional 1 per cent. as a sinking fund to enable such loans to be repayable to the Treasury in a fixed term of years. The 4 per cent. upon such loans representing interest and sinking fund should be made a first charge upon the payments made on behalf of the patients for whose reception the new buildings will be erected.

This plan of finance is practical and simple. It will perpetuate the voluntary principle of hospital management in this country, a result which is momentous for good, for reasons we have fully stated recently in The Hospital. Further, it will protect the national exchequer and ensure that the provision made under the National Insurance Act shall be forthcoming. The State wards in voluntary hospitals would be properly inspected if the Government so wished by their own inspectors; the managers of voluntary hospitals would be relieved from the burden of raising large sums for new buildings and extensions; and in the course of years under this system our nation can be provided with a perfect system of hospital and health provision.

